# CMGC Kinases in Viral Infection and Human Disease

**DOI:** 10.3390/pathogens15040366

**Published:** 2026-03-30

**Authors:** Oluwamuyiwa T. Amusan, Hongyan Guo

**Affiliations:** Department of Microbiology and Immunology, Louisiana State University Health Shreveport, Shreveport, LA 71130, USA

**Keywords:** CMGC kinases, DNA viruses, RNA viruses, DYRK, CDK, p38 MAPK, GSK3, CDKL, RCK, cancer

## Abstract

Cellular processes rely heavily on protein phosphorylation, a mechanism essential for organismal physiology and pathology. The CMGC family comprises a large group of serine/threonine kinases defined by a conserved catalytic core and closely related kinase domains. While several CMGC members have been extensively studied, others, including the RCK and CDKL subfamilies, remain less studied. Here, we synthesize current knowledge of CMGC kinases, emphasizing their structural organization, mechanisms of activation, and roles in infection and disease. CMGC kinases such as CDKs and DYRKs are activated downstream of growth factor signaling to drive proliferative programs. In contrast, other CMGC members respond to cellular stress signals, including stress cytokines, and function during quiescence or adverse conditions to regulate antiproliferative and pro-survival pathways. Through these context-dependent activities, CMGCs govern fundamental cellular processes, including growth, metabolism, transcription, and genome integrity. Although individual CMGC kinases operate within distinct signaling cascades, substantial crosstalk exists among their pathways. Both DNA and RNA viruses exploit host CMGC networks to reprogram the intracellular environment and enhance replication. While CMGC–virus interactions are often proviral, specific CMGC-mediated antiviral responses have been described, notably in SARS-CoV-2 infection. Collectively, CMGC kinases occupy a central position in cellular homeostasis and disease.

## 1. Introduction

Protein phosphorylation is a pivotal process during cellular responses to extracellular and intracellular cues, enabling precise regulation of signaling pathways that control proliferation, differentiation, stress responses, and survival [1]. Reflecting their fundamental importance, protein kinases account for approximately 2% of the human genome [2]. Based on conserved catalytic features, eukaryotic kinases are broadly classified into several major groups, including AGC (PKA/PKG/PKC), CAMK (Ca^2+^/Calmodulin-dependent Protein Kinase), CMGC (Cyclin-dependent kinases [CDKs], Mitogen-activated protein kinases [MAPKs], Glycogen synthase kinases [GSKs], and CDC-like kinases [CLKs]), protein-tyrosine kinase (PTK), and a collection of “other” kinases. With the advent of large-scale sequence datasets, phylogenetic analysis of full catalytic domains further refined this framework, expanding the kinome into eight evolutionary groups [2].

**Figure 1:**
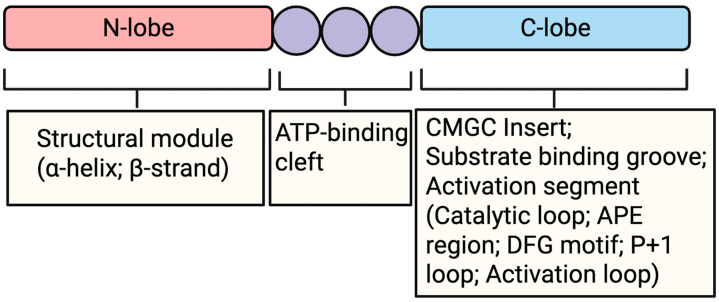
Schematic representation of the structural domains of CMGCs: CMGC kinases share a conserved catalytic core composed of N- and C-lobes separated by an ATP-binding cleft. The N-lobe contains a β-sheet and the regulatory α-helix, which together contribute to kinase activation and structural stability. The C-lobe harbors the substrate-binding groove, activation segment, and the CMGC insert, a defining structural element that mediates interactions with regulatory and scaffolding proteins. The activation segment includes the catalytic loop, APE motif, DFG motif, P + 1 loop, and activation loop, which collectively coordinate catalysis, substrate specificity, and regulatory phosphorylation (Created in BioRender. Amusan, O.T. and Guo, H. (2026) https://app.biorender.com/illustrations/6986076c321a7a8001d9cc26, accessed 12 January 2026).

Among these, the CMGC kinase family comprises approximately 61 human serine/threonine kinases, organized into eight subfamilies, including CDKs, MAPKs, dual-specificity tyrosine phosphorylation-regulated kinases (DYRKs), GSKs, CLKs, serine/arginine-rich protein kinases (SRPKs), tyrosine kinase gene v-ros cross-hybridizing kinase (RCK) kinases, and the relatively unexplored CDK-like kinases (CDKL) [2,3,4]. Despite their diverse biological roles, CMGC kinases share a conserved catalytic architecture consisting of N- and C-lobes flanking an ATP-binding cleft, as well as a defining CMGC insert within the C-lobe that serves as a scaffold for regulatory protein–protein interactions (Figure 1) [5]. Functional specificity within this family is achieved through three integrated layers: (i) activation mechanisms, such as cyclin binding, dual-phosphorylation, or autophosphorylation; (ii) substrate recognition, often centered on proline-directed motifs coordinated by the P + 1 loop; and (iii) docking interactions mediated by auxiliary motifs or binding partners. Although many CMGC kinases preferentially phosphorylate serine/threonine residues within S/T-P motifs, notable exceptions underscore the functional specialization within this group [5,6]. Hence, CMGC kinases operate as integrative signal hubs, coordinating cell cycle progression, transcription, RNA processing, stress responses, and cell fate decisions.

In this review, we synthesize current understanding of CMGC kinases by focusing on their shared structural organization, comparative regulatory logic, and extensive signaling crosstalk. We place particular emphasis on how these properties are exploited in host–pathogen interactions and disease states, and how the integrative nature of CMGC signaling shapes both physiological homeostasis and pathological outcomes.

### 1.1. Structural and Functional Organization of the CMGC Kinases

#### 1.1.1. Cyclin-Dependent Kinases (CDKs)

CDKs comprise approximately 20 members that are often grouped into canonical cell cycle CDKs (e.g., CDK1, CDK2, CDK4, CDK6) and non-canonical CDKs with prominent roles in transcription, RNA processing, neuronal function, and differentiation (e.g., CDK5, CDK7–13, CDK14–20). Structurally, CDKs conform to the conserved CMGC catalytic architecture, with N- and C-lobes flanking and an ATP-binding cleft. Unlike most other CMGC kinases, CDKs are catalytically inactive as monomers and rely on cyclin binding as an obligate activation step. Cyclin association stabilizes the αC helix and repositions the activation segment within the C-lobe, enabling phosphorylation of the activation loop threonine (Thr160/161) by the CDK-activating kinase (CAK). Dephosphorylation of this site serves as a key inhibitory mechanism, highlighting how CDK activity is tightly regulated by both phosphorylation and protein–protein interactions [7,8].

Most CDKs preferentially phosphorylate S/T-P motifs, but substrate selection is shaped less by minimal peptide consensus than by cyclin-mediated recruitment, docking surfaces, and multisite context. This mode of regulation distinguishes CDKs from docking-motif–dependent CMGC kinases such as MAPKs. Many CDK substrates, including the retinoblastoma protein (Rb), are nuclear, and several CDKs are constitutively nuclear or undergo cell cycle-regulated nuclear import [9]. In some cases, nuclear localization is conferred by associated cyclins or intrinsic nuclear localization sequences, whereas other CDKs remain predominantly cytoplasmic [10,11], reflecting functional diversification within the family.

#### 1.1.2. Dual-Specificity Tyrosine Phosphorylation-Regulated Kinases (DYRKs)

DYRKs comprise five members (DYRK1A, DYRK1B, DYRK2, DYRK3, and DYRK4) and are subdivided into class I (DYRK1A/1B) and class II (DYRK2/3/4) based on sequence features and tissue expression patterns. While class I DYRKs are widely expressed across major tissues such as the adrenal gland and the cervix, class II DYRKs have more restricted tissue expression [12,13]. All DYRKs share a central kinase domain flanked by regulatory N- and C-terminal regions but are distinguished from other CMGC kinases by their maturation-dependent activation mechanism. DYRKs undergo autophosphorylation on a conserved tyrosine residue within the activation loop, a process facilitated by the DYRK homology box located N-terminal to the kinase domain. This autophosphorylation event “licenses” subsequent serine/threonine kinase activity, rendering DYRKs constitutively active once matured.

Class I DYRKs contain C-terminal proline-, glutamate-, serine-, and threonine-rich (PEST) motifs that promote protein turnover via E3 ubiquitin ligase recruitment [14]. Class II DYRKs possess an N-terminal autophosphorylation accessory (NAPA) region that is critical for efficient activation-loop Tyr autophosphorylation and full catalytic activation [15]. Additional family-specific features, such as histidine- and serine/threonine-rich repeats in DYRK1A, further modulate subcellular localization and catalytic output.

Most DYRKs contain nuclear localization sequences and shuttle between the cytoplasm and nucleus, consistent with roles in chromatin regulation, DNA damage responses, and transcription [16]. DYRK3 represents an exception, localizing to both compartments and stress granules, highlighting functional diversification within the family [17].

#### 1.1.3. Mitogen-Activated Protein Kinases (MAPKs)

MAPKs translate extracellular cues into intracellular responses through hierarchical kinase cascades. The conventional MAPKs, including extracellular signal-regulated kinases 1/2 (ERK1/2), c-Jun N-terminal kinases 1–3 (JNK1/2/3), p38 MAPKs (α, β, γ, δ), and ERK5, share a conserved threonine-X-tyrosine (TXY) motif in the activation loop. Dual phosphorylation of the threonine and tyrosine residues in this motif by upstream MAPKs (MKKs) induces conformational changes that align catalytic residues and stabilize the active state. In contrast, atypical MAPKs are defined by non-canonical activation-loop motifs and/or regulatory logic: ERK3/4 replace TXY with SEG, Nemo-like kinase (NLK) contains a TQE motif, whereas ERK7/8 retains a TEY motif but is regulated atypically [18].

Although MAPKs are proline-directed, substrate specificity is dictated primarily by stable docking interactions rather than minimal peptide consensus. MAPKs engage substrates, upstream kinases, and phosphatases through dedicated docking motifs (e.g., D-sites and DEF motifs) located outside the catalytic cleft, distinguishing them from cyclin-dependent CDKs and priming-dependent GSKs. These interactions also influence MAPK subcellular localization, enabling spatially restricted signaling. This modular docking-based architecture allows MAPKs to coordinate stress responses, inflammation, differentiation, and development with high fidelity.

#### 1.1.4. Glycogen Synthase Kinases (GSKs)

GSKs represent a distinct subgroup within the CMGC family characterized by their reliance on primed substrates. The GSK family is composed of 1–5, and GSK3 has been extensively studied with increasing physiological significance. In mammals, the GSK subgroup is represented primarily by two closely related paralogs, GSK-3α and GSK-3β. Both share a highly conserved kinase domain and are constitutively phosphorylated on activation-loop tyrosines (Y279 in GSK-3α and Y216 in GSK-3β), conferring basal catalytic activity under steady-state conditions [19]. Unlike most CMGC kinases, GSKs are active in the absence of upstream stimulation and are regulated predominantly through inhibitory phosphorylation. Phosphorylation of N-terminal serines (S21 in GSK-3α and S9 in GSK-3β) by kinases such as Akt suppresses activity by interfering with substrate binding [20,21]. GSKs exhibit a unique priming requirement, preferentially phosphorylating serine or threonine residues positioned four amino acids upstream of a pre-phosphorylated site (S/T-X-X-X-S/T(P)) [21]. This primed-substrate recognition mechanism enables GSKs to function as signal integrators that act downstream of other kinase pathways, a property exploited in developmental signaling and viral infection. GSK-3β localizes predominantly in the cytoplasm but can translocate to the nucleus and mitochondria via karyopherin-mediated transport [22,23], whereas GSK-3α is often excluded from the nucleus through N-terminal regulatory mechanisms, underscoring isoform-specific functions [24].

#### 1.1.5. CDC-like Kinases (CLKs)

CLKs (CLK1–4) are evolutionarily conserved kinases specialized for RNA processing. They contain a canonical CMGC kinase domain with a characteristic EHLAMMERILG (“LAMMER”) motif in the C-lobe, along with MAPK-like insertions proposed to contribute to substrate recognition [25]. CLKs are regulated by phosphorylation and growth-factor-responsive pathways (including Akt in some contexts) to modulate CLK activity and/or distribution, linking splicing control to cellular signaling [26,27]. CLKs preferentially phosphorylate serine/arginine-rich (RS) domains with R-x-x-S/T motifs, dynamically regulating spliceosome assembly and alternative splicing. Although CLKs shuttle between the cytoplasm and nucleus, their primary functions are nuclear, where they control splicing factor activity [28,29].

#### 1.1.6. Serine/Arginine-Rich Protein Kinases (SRPKs)

SRPK 1, 2, and 3 comprise the SRPK family. SRPKs share a conserved kinase domain interrupted by a large spacer region and a C-terminal RS domain. The spacer region plays a critical role in subcellular localization and protein–protein interactions. SRPKs are often retained in the cytoplasm through interactions with chaperones and anchoring factors associated with these regions. Upon signaling-dependent remodeling and/or phosphorylation, SRPKs phosphorylate RS-domain-containing splicing factors to promote nuclear import and modulate spliceosome dynamics [30,31]. CLKs and SRPKs thus operate coordinately to couple signaling cues to RNA splicing decisions.

#### 1.1.7. ROS-Cross-Hybridizing Kinases (RCKs)

The RCK family comprises MAK, ICK, and MOK and represents a structurally hybrid subgroup within the CMGC kinase family. RCK members harbor a TDY motif in the activation loop that combines features of the TXY motif of MAPKs and the tyrosine-regulated activation of CDKs. Activation of MAK and ICK requires two phosphorylation events: autophosphorylation of the tyrosine within the TDY motif, followed by phosphorylation of the threonine by the CDK-related kinase CCRK. This dual mechanism positions RCKs as integrators of intrinsic and extrinsic regulatory signals [32,33]. MAK is predominantly nuclear but relocalizes to mitotic structures during cell division, where it restrains mitotic progression. In contrast, the biological roles and regulation of MOK remain poorly characterized, highlighting a major knowledge gap.

#### 1.1.8. CDK-like Kinases (CDKL)

The CDKL family (CDKL1-5) represents the least characterized CMGC subgroup. CDKL5 is the best studied owing to its association with developmental and epileptic encephalopathies. Structurally, CDKL5 shares features with classical CDKs, including an ATP-binding domain, a serine/threonine kinase active site, and a putative cyclin-binding region. CDKL5 contains a TEY motif within the activation loop that undergoes autophosphorylation, suggesting a MAPK-like activation mechanism. The presence of both nuclear localization and nuclear export signals indicates dynamic nucleocytoplasmic shuttling, consistent with roles in transcriptional and epigenetic regulation [22].

### 1.2. CMGC Signaling and Cross-Talk Among CMGC Kinases

**Figure 2:**
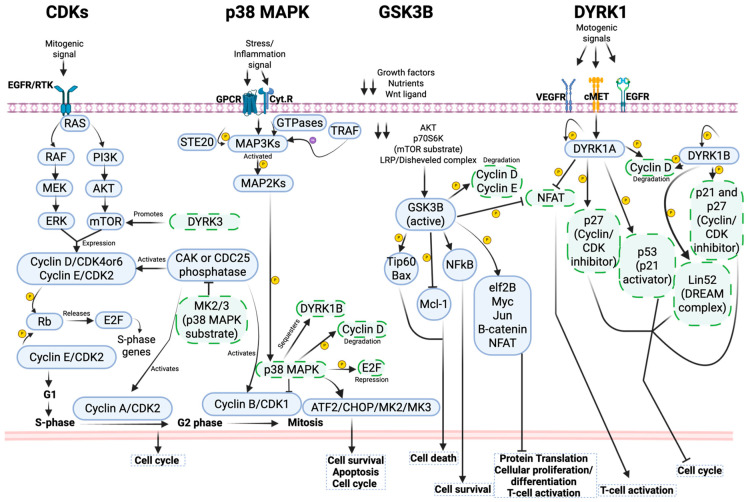
A schematic diagram illustrating the signaling (blue undashed boxes) and signaling crosstalks (green dashed boxes) of selected CMGC kinases. Mitogenic signals, stress/inflammation signals, decreases in growth factors, or motogenic signals can activate CDKs, p38 MAPK, GSK3Β, and DYRK1, respectively. Upon ligand-receptor interaction, cyclin/CDK complexes are synthesized via the RAF-ERK or AKT-mTOR pathways, which then trigger a cascade leading to cell cycle regulation. The activities of these cyclin/CDK complexes can be inhibited by the p38 MAPK pathway and promoted by DYRK or CAK/CDC25. In the p38 MAPK pathway, ligand-receptor interaction activates upstream MAP3Ks kinases either through direct phosphorylation, ubiquitination, or adaptor interaction. These kinases eventually activate p38 MAPKs. When activated, p38 MAPK promotes the activity of transcription factors that influence cell survival, apoptosis, or the cell cycle. p38 MAPK can also modulate cell cycle factors and DYRK activity. For GSK3Β, a decrease in the AKT/p70S6K/LRP/Disheveled complex releases GSK3Β in its active form, which then activates factors that induce cell death, survival, or inhibit specific cellular processes. This kinase can also directly induce the degradation of certain cyclins. Upon ligand-receptor binding, DYRK1 is activated, promoting the activation of specific cell cycle inhibitors. DYRK1 can also phosphorylate and inhibit NFAT, a GSK3Β substrate. This diagram highlights the potential crosstalk among CMGC kinases or their substrates, emphasizing CMGCs’ functional overlap (Created in BioRender. Amusan, O.T. and Guo, H. (2026) https://app.biorender.com/illustrations/6923c9e8b5b50dbc7ee5f618, accessed 12 January 2026).

Although CMGC kinases are often described within discrete signaling cascades, their shared structural similarities and overlapping substrate space position them within a highly interconnected regulatory network (Figure 2). Rather than functioning as isolated modules, CMGC members continually influence one another’s activity, collectively integrating mitogenic, metabolic, and stress signals to determine cell fate. Nowhere is this more evident than in the coordination of cell cycle progression with environmental status.

Mitogenic cues such as growth factors and nutrients engage receptor tyrosine kinases (RTKs) and activate RAS-dependent RAF–MEK–ERK and PI3K–AKT–mTOR pathways. These pathways converge on the induction and assembly of Cyclin D/CDK4 or CDK6 complexes, which translocate to the nucleus and are activated by CAK and CDC25 phosphatases. Activated CDK4/6 phosphorylate retinoblastoma protein (Rb), releasing E2F transcription factors and inducing S-phase genes. ERK- and mTOR-dependent signaling subsequently promotes Cyclin E/CDK2 activation, driving the G1–S transition. During the S phase, Cyclin E/CDK2 and Cyclin A/CDK2 maintain Rb hyperphosphorylation to sustain DNA synthesis, while Cyclin A/CDK2 prevents re-replication by inhibiting primase. Entry into mitosis is then orchestrated by sequential activation of Cyclin A/CDK2 and Cyclin B/CDK1, culminating in chromosome condensation, spindle assembly, and mitotic progression (Figure 2). This cascade exemplifies how CDKs encode temporal order, each step establishing the biochemical conditions required for the next.

Stress-responsive CMGC kinases counterbalance this proliferative drive. Canonically, the p38 MAPK pathway is activated through cytokine and GPCR receptors. When these receptors respond to their stress-/inflammation-induced signals, MAP3Ks are activated via three ways: MAP3Ks’ interactions with small GTPases such as RHO, CDC42, and RAC; through phosphorylation by STE20 kinases, or via ubiquitylation mediated by TRAF ubiquitin ligases [34]. Upon activation, MAP3K members such as ASK1, DLK, MEKK3, MEKK4, and MLK3 phosphorylate and activate MAP2Ks. The latter kinase then phosphorylates and activates p38 MAPK [34]. Although this canonical pathway occurs in various cell types, an atypical pathway has been observed in cardiomyocytes and T cells. In this non-canonical pathway, the autophosphorylation of p38α, a subunit of the p38 MAPK, is triggered either through its association with transforming growth factor-β-activated protein 1 (TAB1) [35] or by direct phosphorylation mediated by ZAP70, which is a downstream kinase of T cell receptor (TCR) activation [36].

Once activated, p38 MAPK phosphorylates transcription factors (e.g., ATF2, CHOP) and effector kinases (MK2/3) that reshape gene expression and cellular behavior [37]. A central function of p38 signaling is to restrain inappropriate proliferation. For example, MK2/3 inhibit CDC25 phosphatases, which are key activators of cyclin/CDKs, to halt cell progression [38]. Similarly, p38 MAPK can activate HBP, p16, and p19, which are inhibitors of Cyclin D/CDK4/6 and Cyclin E/CDK2, or it can phosphorylate cyclin D directly to promote its degradation [39] and block the G1-S transition [40]. Although it is well known that CDKs often phosphorylate RB to release E2F for transcriptional activation, p38 MAPK can also independently phosphorylate E2F and cause its repression [41]. In addition, p38 activity has been linked to inhibition of G2 progression through the suppression of Cyclin B/CDK1 activity [42]. Through these mechanisms, p38 establishes a stress-imposed “brake” on the CDK engine, preserving tissue integrity under adverse conditions.

GSK3 further embeds environmental status into CMGC logic. GSK3 signaling is typically activated during the cellular resting phase, when growth factors, nutrients, or the Wnt signals are absent. Under these conditions, inhibitors of GSK3, such as AKT, p70S6K (an mTOR substrate), or the LRP/Disheveled complex, remain at low levels, so that GSK3 is activated [43,44]. Active GSK3 suppresses translation and proliferation by targeting eIF2B, Myc, NFAT, Jun, and β-catenin, while also modulating survival pathways through NF-κB and apoptotic regulators. Upon activation, GSK3 phosphorylates various transcription factors and effector proteins that regulate cell death and survival. Specifically, active GSK3 can suppress eIF2B, Myc, NF-AT, Jun, and β-catenin to inhibit protein translation, cellular proliferation, T cell activation, and overall cellular responses [44]. Interestingly, based on the cellular context, GSK3 has also been shown to promote cell survival through NF-κB activation or to induce cell death through inhibition of the anti-apoptotic protein Mcl-1 and the activation of pro-apoptotic proteins such as Tip60 and Bax [45,46]. GSK3 also crosstalks with Cyclin/CDKs; it phosphorylates cyclins D and E, targets them for degradation, and inhibits the G1–S transition of the cell cycle [44], consistent with the antiproliferative functions of GSK3. Thus, mitogenic pathways that activate CDKs simultaneously silence GSK3, whereas nutrient-poor or stress states invert this balance, thus illustrating how CMGC members respond to environmental polarity.

DYRKs extend this regulatory web. Beyond cell cycle control, DYRKs influence transcription, DNA damage responses, and developmental signaling (e.g., Hedgehog, Notch, Wnt) [12]. DYRK1A integrates growth-factor inputs from VEGFR, cMET, and EGFR and can suppress G1 progression by destabilizing Cyclin D1, activating p27 and p53, or engaging the DREAM complex [12,47]. DYRK1B mirrors these functions under stress [48,49,50,51], while DYRK2 coordinates DNA damage responses through CDC25A degradation [52] and p53 activation [53], yet also constrains oncogenic transcription factors such as c-Myc and c-Jun [54]. In contrast, DYRK3 enhances mTOR activity, favoring Cyclin D/CDK4/6 assembly and proliferative entry [17]. Although poorly characterized, DYRK4 appears to contribute to genome integrity [55]. Crucially, DYRKs do not operate in isolation. p38 MAPK can sequester and suppress DYRK1B [56], while DYRK1A phosphorylates and inactivates GSK3β, thereby releasing β-catenin signaling and altering metabolic and proliferative outcomes [57]. These reciprocal interactions illustrate that CMGC kinases regulate not only shared substrates but also one another, forming feedback-rich circuits rather than linear hierarchies.

Collectively, CMGC kinases constitute an integrated signaling framework that couples growth, stress, metabolism, transcription, and genome integrity (Figure 2). Proliferation is not simply “on” or “off”; it emerges from the dynamic balance between CDK-driven progression and the countervailing forces of p38 MAPK, GSK3, and DYRKs. Viewing CMGCs as a network, rather than as parallel pathways, provides a more accurate conceptual model for how cells compute fate decisions. Dissecting this crosstalk will be essential for identifying central regulatory nodes whose modulation could recalibrate entire disease networks in cancer, neurodegeneration, and infection.

### 1.3. CMGC Kinases in Viral Infections

Viruses are obligate intracellular parasites and must repurpose host signaling networks to complete their life cycles. CMGC kinases, positioned at the nexus of cell cycle control, transcription, stress responses, and RNA processing, are therefore frequent viral targets. Rather than acting as passive substrates, these kinases function as regulatory “switchboards” that viruses tune to reprogram cellular state. The outcome of CMGC engagement is highly context-dependent, shaped by viral genome type, cell types, and the stage of infection. Here, we group viruses into two major classes based on genome type: DNA viruses and RNA viruses. We summarize interactions between selected CMGC kinase family members and representative DNA or RNA viruses, with an emphasis on the functional outcomes of these interactions (Table 1).

### 1.4. CMGCs and DNA Viruses

DNA viruses possess DNA genomes that serve as templates for both viral DNA replication and viral gene transcription, processes that, except for Poxviruses, occur in the nucleus. Following viral entry, the capsid is released into the cytoplasm, undergoes uncoating, and the viral genome is subsequently transported into the nucleus through the nuclear pore complex. Successful nuclear delivery and replication require evasion or modulation of intrinsic cellular sensing and antiviral defense mechanisms. Once in the nucleus, early viral gene products are transcribed by viral and/or host transcriptional machinery, including host RNA polymerase II. These early proteins establish a permissive environment for viral DNA replication through direct or indirect interactions with both the viral genome and the host cell [208]. Small DNA viruses with circular double-stranded DNA genomes, such as papillomaviruses and polyomaviruses, rely primarily on host DNA polymerases for genome replication. In contrast, larger DNA viruses, including herpesviruses and poxviruses, encode their own DNA polymerases; nevertheless, inhibition of host DNA polymerases can still impair herpesvirus replication [209,210]. Thus, both viral and host DNA polymerases are critical determinants of DNA virus replication efficiency.

Another essential requirement for viral DNA replication is the availability of primers, which are typically generated by cellular primase. Most DNA viruses depend on host primase activity for primer synthesis, with notable exceptions such as adenoviruses [211]. Equally important is access to sufficient intracellular deoxynucleotide (dNTP) pools, which DNA viruses largely derive from host cellular metabolism [212]. Once these conditions are met, newly synthesized viral DNA serves as a template for intermediate and late viral gene expression. As with host mRNAs, the cellular splicing machinery is often required to generate mature viral transcripts that are competent for translation [213]. Consequently, DNA viruses are under strong selective pressure to manipulate host cell cycle and transcriptional programs, often by targeting CMGC kinases that govern these processes.

CDKs are among the most frequently co-opted CMGC members. Small DNA oncogenic viruses, such as human papillomaviruses (HPVs), synchronize viral genome replication with host cell cycle progression by directly promoting cyclin-CDK complex formation (e.g., Cyclin D1/CDK4, Cyclin A/CDK2, and Cyclin B/CDK1) or by suppressing endogenous CDK inhibitors [58,59,60]. In contrast, large DNA viruses, including herpesviruses, encode their own replication machinery and therefore exhibit greater flexibility [209]. Herpesviruses can either stimulate or restrain CDK activity depending on infection stage and cellular context: HCMV upregulates cyclin H within the CAK complex [62], whereas HSV-1 and EBV encode proteins that suppress S-phase cyclins or induce CDK inhibitors [65,73]. This bidirectional control strengthens the possibility that viruses do not simply “activate” or “inhibit” CDKs, but dynamically tune cell cycle and transcriptional states to optimize viral gene expression. Beyond CDKs, DYRK family members emerge as broadly proviral factors for DNA viruses. Pharmacological inhibition of DYRKs exhibits anti-herpesviral activity [83], and multiple DNA viruses actively upregulate DYRK expression or function. HPV and HCMV increase DYRK1A or DYRK1A/B levels [82,84], while hepatitis B virus (HBV) recruits DYRK1A to its covalently closed circular DNA (cccDNA) to enhance viral transcription [85]. These observations suggest that DYRKs provide a conserved regulatory axis through which DNA viruses amplify nuclear transcriptional capacity.

Stress-responsive CMGC kinases are similarly co-opted. The p38 MAPK pathway is activated by many DNA viruses, including HCMV, KSHV, EBV, and HSV, and is often required for efficient replication, likely by coupling stress signaling to survival and transcriptional programs [88,89,90,91,92,94,95,96]. GSK3β illustrates the duality of CMGC function in infection: although GSK3β can promote antiviral signaling via IRF3 or NF-κB, viruses frequently repurpose its activity for assembly or growth control [214]. Simian cytomegalovirus uses GSK3β to phosphorylate capsid assembly proteins [99], while KSHV manipulates GSK3β–β-catenin signaling to drive host proliferation [215]. In neurons, HSV-induced GSK3β activation contributes to synaptic dysfunction, linking viral CMGC modulation to long-term neuropathology [102,103].

Although many DNA viruses do not universally use alternative splicing to expand coding capacity from individual transcripts, RNA splicing remains essential for the efficient maturation of specific viral mRNAs and proper viral gene expression [213]. Accordingly, DNA viruses target splicing-associated CMGC kinases, particularly SRPKs and CLKs. HPV depends on SRPK1 for productive expression of viral transcripts, and viral proteins can sequester or redirect SRPKs to bias RNA processing [108,110]. VZV and EBV exploit SRPKs to enhance viral mRNA export and lytic replication [114,115], while HBV uses SRPK1/2 to phosphorylate core proteins and facilitate capsid assembly [116,118,119]. HSV-1 further demonstrates the strategic flexibility of this axis: its ICP27 protein relocalizes SRPK1 to suppress host splicing in favor of viral mRNA translation [112,113]. CLKs are similarly activated during HBV infection, and their inhibition reduces intracellular cccDNA, highlighting splicing kinases as unexpected regulators of viral persistence [107]. Collectively, these examples reveal a unifying principle: DNA viruses reshape the “nuclear economy” of infected cells by rewiring CMGC kinase networks. Through selective activation or suppression of CDKs, DYRKs, MAPKs, GSKs, SRPKs, and CLKs, viruses coordinate cell cycle state, transcriptional output, stress adaptation, and RNA processing to favor viral replication. CMGC kinases thus serve not merely as viral substrates, but as integrative control nodes through which pathogens reprogram host cell identity.

### 1.5. CMGC Kinases and RNA Viruses

Unlike DNA viruses, RNA viruses rely on virally encoded RNA-dependent RNA polymerases (RdRps) to synthesize both genomic RNA and mRNA. Following entry and uncoating, positive-sense RNA genomes are immediately translated, whereas negative-sense RNA genomes must first be transcribed by RdRp using host nucleotide pools. As infection progresses, viral programs shift from mRNA production to genome replication, imposing escalating demands on cellular metabolism, translation, and RNA-handling machinery [216]. Productive RNA virus infection, therefore, depends on maintaining a permissive cellular state, the one that sustains nucleotide availability, protein synthesis, stress tolerance, and, in many cases, transcriptional plasticity. CMGC kinases, which govern precisely these processes, emerge as central regulatory nodes.

A recurrent strategy among RNA viruses is the manipulation of cell cycle-associated CDKs. Viruses such as SARS-CoV-2, Influenza virus, HTLV, and HCV modulate S-phase entry or progression by either inhibiting or activating appropriate cyclin/CDK complexes [120,121,124] (Table 1). These interventions are not uniform: some viruses suppress S-phase entry, whereas others transiently stimulate proliferative signals. This may arise either as a result of the virus’s quest to prevent premature cell death, evade immune detection, facilitate assembly, or ensure that resources are available for maximal genome replication/progeny production. Influenza virus, for example, drives cells toward a G0/G1-like state [124], even though the inhibition of CDK1 or CDK9 suppresses viral replication, revealing that discrete CDK activities remain essential even within a globally arrested cell [125,126]. Cyclin D3 can restrict the incorporation of SARS-CoV-2 envelope into virions [123], which may explain why the virus restricts cyclin D in certain contexts [120]. Reoviruses exemplify a more aggressive strategy, broadly inhibiting CDKs to impose a stress-like state that favors viral production [217]. Avian reovirus p17 directly targets CDK1, CDK2, CDK4, and CDK6 and represses CAK activity by promoting p53/cyclin H interaction [127]. These contrasting examples underscore a central principle: RNA viruses do not simply “halt” or “activate” the cell cycle; instead, they sculpt specific CDK outputs to balance host survival with optimal viral yield.

Stress-responsive CMGC kinases are even more uniformly exploited. Virtually all RNA viruses surveyed activate p38 MAPK, and blockade of this pathway suppresses replication in most settings (Table 1). This reliance reflects the dual role of p38 signaling in coordinating stress adaptation, survival, and transcriptional reprogramming, conditions favorable for sustained viral RNA synthesis. GSK3β similarly acts as a proviral effector: it directly phosphorylates viral structural proteins from diverse RNA viruses, including PEDV, SHVV, and SARS-CoV-2, thereby enhancing assembly or replication [163,164,167]. DYRKs, although less universally engaged, are repurposed by coronaviruses in distinctive ways. Rather than driving cell cycle outputs, DYRK1A is exploited to enhance receptor expression and chromatin accessibility, aligning nuclear transcriptional landscapes with viral needs [181,182]. The localization of DYRK1A, primarily in the nucleus, and its role in chromatin remodeling may account for this virus-induced advantage.

Retroviruses and other integrating RNA viruses impose an additional layer of dependence on host transcriptional machinery. HIV exemplifies precise hijacking of transcriptional CMGCs. The viral Tat protein co-opts CDKs that phosphorylate the RNA polymerase II C-terminal domain, thereby accelerating transcriptional elongation from the proviral genome [186,187,189]. Tat also engages splicing-associated CDKs, including CDK13, to optimize viral RNA processing [190]. In parallel, inhibition of CDK2 or CDK6 stabilizes SAMHD1, reducing intracellular dNTP pools and impairing HIV replication, highlighting how CDKs intersect with metabolic control to regulate viral fitness [188]. In contrast, HTLV favors long-term persistence and oncogenesis by broadly stimulating cyclin/CDK activity through its Tax protein, reinforcing proliferative circuits rather than finetuning transcriptional elongation [194,195,218].

RNA viruses also converge on CMGC kinases that govern RNA maturation. SRPKs and CLKs are repeatedly identified as proviral across diverse RNA viruses, including HCV, HIV, and SARS-CoV-2, with pharmacological inhibition suppressing viral replication [196,197,199]. SRPK2 is upregulated in COVID-19 patients, underscoring its relevance in vivo [198]. Beyond splicing, these kinases can directly modify viral proteins: SRPK1 phosphorylates Ebola virus VP30 to promote transcription [201], while CLK1 is required for the generation of the spliced M2 transcript of influenza virus [204,205,206]. These examples reveal that RNA viruses do not merely borrow host RNA-processing machinery; they actively reprogram it through CMGC kinases.

Together, RNA viruses exploit CMGC kinases to choreograph a cellular environment optimized for RNA synthesis, protein production, and stress tolerance. Rather than applying a single strategy, they differentially prioritize cell cycle control, stress signaling, transcription, or RNA processing according to viral lifestyle and cellular context. CMGC kinases thus function as programmable control points through which RNA viruses reshape host cell identity. Deciphering how these interactions are dynamically tuned provides a conceptual framework for host-directed antiviral strategies targeting shared regulatory hubs rather than individual viral components.

### 1.6. CMGCs in Other Human Diseases

#### 1.6.1. CDKs: Context-Dependent Oncogenes and Systemic Regulators

**Figure 3:**
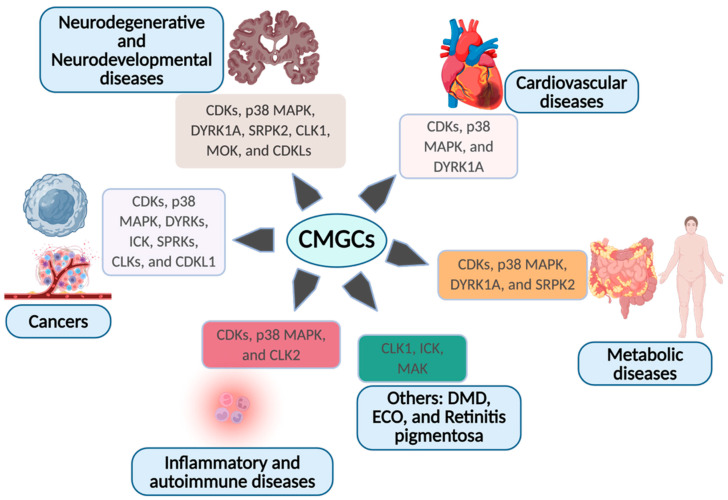
A schematic diagram illustrating the role of CMGC kinases in human diseases. All the discussed CMGC kinase groups can contribute to neurodegenerative diseases and cancers, while other disease effects are specific to certain groups (Created in BioRender. Amusan, O.T. and Guo, H. (2026) https://app.biorender.com/illustrations/6928a4a432d2a4f6f455ffca, accessed 12 January 2026).

The same properties that make CMGC kinases powerful integrators of cellular state in infection, enabling them to control proliferation, transcription, stress responses, and RNA processing, also position them as central determinants of organismal homeostasis and disease. Dysregulation of CMGC signaling reverberates across tissues, manifesting most prominently in cancer, neurodegeneration, and metabolic and cardiovascular disorders (Figure 3). A striking theme is convergence: distinct CMGC subfamilies, despite their mechanistic diversity, are repeatedly implicated in overlapping disease spectra. The role of CDKs in cancer is paradigmatic. Aberrant activation of cell cycle CDKs drives uncontrolled proliferation, genomic instability, and epigenetic reprogramming [219,220]. Upregulation of CDK1 or CDK2 perturbs mitotic fidelity, fostering chromosomal instability [221,222], while CDK4/6 can override mitogenic and antiproliferative checkpoints [223]. CDKs also rewire transcriptional landscapes as CDK4 activates PRMT5 to reshape chromatin [224], and transcriptional CDKs such as CDK7 and CDK9 amplify oncogene expression [219,225,226]. Beyond promoting cancer through cell cycle and transcriptional regulation, CDKs can enhance stemness, angiogenesis, and metabolism [219]. For instance, CDK6, independent of cyclin D, can upregulate VEGFA to promote angiogenesis [227]. These mechanisms underpin the widespread clinical deployment of CDK inhibitors [219].

Yet CDKs are not unidimensional oncogenes. In specific contexts, CDK1, CDK4, CDK5, CDK10, CDK11, and CDK12 exhibit tumor-suppressive properties [219], reflecting an intrinsic tension between proliferation and viability. Excessive CDK1 activity, for instance, can induce catastrophic mitosis and telomere attrition, enforcing arrest rather than growth [219,228,229]. Other CDKs restrain oncogenic programs by suppressing TGFβ signaling [230] or epithelial–mesenchymal transition [231]. Thus, CDKs embody a duality: they can fuel malignancy, yet their overactivation may destabilize the very cellular systems cancers depend upon. Beyond oncology, CDKs shape systemic disease. CDK5 is central to neuronal development and neurodegeneration [232], while CDK2 and multiple transcriptional CDKs are linked to Alzheimer’s disease [233,234,235,236,237]. In the heart, CDK1 and CDK4 regulate cardiomyocyte proliferation and influence susceptibility to myocardial injury [238]. CDK4/6 couples metabolic activation to adipogenesis and β-cell expansion, intersecting with obesity and diabetes [239,240]. In autoimmune disease, CDKs drive pathogenic fibroblast activation in rheumatoid arthritis [219,241,242,243]. These pleiotropic roles highlight CDKs as global regulators of tissue identity.

#### 1.6.2. p38 MAPK and DYRKs: Stress, Degeneration, and Metabolism

p38 MAPK exemplifies how stress-responsive CMGC kinases bridge physiology and pathology. Essential for neuronal excitability and glial function, p38α also mediates neuronal hypersensitivity and degeneration [244,245]. Elevated p38α activity accompanies Alzheimer’s disease, ALS, and Parkinson’s disease [34,246], and p38 inhibitors are actively explored therapeutically [247]. In the cardiovascular system, p38 suppresses cardiomyocyte proliferation by repressing NFAT, contributing to hypertrophy and heart failure [248,249]. Through transcription factors such as MEF2, CREB, and PPARs, p38 also governs metabolic reprogramming in obesity [250,251].

DYRKs mirror and extend these themes. DYRK1A, encoded within the Down syndrome critical region [252], is exquisitely dosage sensitive: both overexpression and insufficiency disrupt neurodevelopment [253,254,255]. DYRK1A suppresses pancreatic β-cell proliferation [256], contributing to diabetes, and restrains cardiomyocyte renewal via cyclin D degradation [257]. Across cancers, DYRKs regulate cell cycle checkpoints, DNA damage responses, and oncogenic signaling [12,256]. DYRK2 is frequently amplified in tumors, DYRK1B promotes tumor progression, and DYRK1A exhibits context-dependent tumor-suppressive or oncogenic behavior [12,258,259,260]. Even the understudied DYRK3 and DYRK4 show altered expression across malignancies [12]. Together, DYRKs recapitulate the central CMGC paradox that is essential for homeostasis but hazardous when dysregulated.

#### 1.6.3. RNA Processing Kinases: SRPKs and CLKs

Splicing-associated CMGC kinases further broaden disease impact. With the exception of SRPK4, dysregulation of SRPKs is linked to tumor initiation and progression [261,262]. SRPK2 has emerged as a driver of metabolic liver disease, and its inhibition ameliorates alcohol-associated liver pathology in mice [263]. Enriched expression of SRPK2 in the brain implicates it in neurodegeneration [264,265]. CLKs occupy a similarly expansive disease space: they contribute to cancer, neurodegeneration (notably CLK1), inflammatory disease (CLK2), muscular dystrophy, and autophagy-associated disorders (reviewed in [25]). These kinases illustrate how perturbation of RNA-processing hubs can propagate systemic pathology.

#### 1.6.4. RCKs and CDKLs: Emerging Disease Axes

RCKs (MAK, ICK/CILK1, and MOK) govern ciliogenesis, spermatogenesis, and Hedgehog signaling [266]. MAK mutations underlie retinitis pigmentosa, linking CMGC dysfunction to sensory degeneration [267]. ICK mutations cause endocrine–cerebro–osteodysplasia (ECO) syndrome and are associated with colorectal cancer [268,269]. MOK remains enigmatic, but recent evidence implicates it in ALS via regulation of microglial inflammation [270]. CDKL kinases, though least characterized, already define a major neurodevelopmental axis: CDKL5 mutations cause developmental and epileptic encephalopathy [271,272], and emerging variants in CDKL1 and CDKL2 associate with intellectual disability and developmental delay [273]. CDKL1 overexpression has also been linked to colorectal tumor growth [274].

## 2. Conclusions and Future Perspectives

CMGC kinases occupy a central position in governing essential cellular processes and shaping physiology and disease. Although many members have been extensively investigated, several remain largely unexplored. This includes transcriptional CDKs such as CDK14, CDK15, and CDK20. Given the shared sequence features, subcellular localization, and activation mechanisms across CDKs, existing knowledge can be leveraged to guide systematic interrogation of these understudied kinases and to define their roles in cell biology and diseases. Moreover, while embryonic deletion of CDK1 is known to be lethal [275], the precise functions of CDK1 and other CDKs in embryogenesis and tissue homeostasis remain poorly defined. Tissue-specific knockout strategies, as previously done by Diril et al. (2012) [276], combined with advanced imaging and functional genomics, offer powerful avenues to address these gaps.

Beyond CDKs, other CMGC members, including CDKLs, DYRK3, DYRK4, and MOK, are also underexplored. Although CDKL proteins have been implicated in histone remodeling, microtubule trafficking, cell polarity, and transcriptional regulation, the underlying mechanisms remain largely undefined. As compared with DYRK1A and DYRK1B, which are strongly linked to cancer, DYRK3 and DYRK4 have received little attention. Given their robust transcript expression in the testis relative to other tissues [12], these kinases may play specialized roles in testicular physiology or pathology. Emerging evidence further implicates MOK in amyotrophic lateral sclerosis through the promotion of inflammatory responses in microglia [270], and elevated MOK expression correlates with metabolic disorders such as type 1 diabetes mellitus [277,278]. Whether MOK-driven inflammation extends to other disease contexts remains an open and important question.

Most viruses enhance CMGC kinase activity, although some repress it, underscoring the broader conclusion that viruses co-opt these kinases for their own benefit (Table 1). The interaction between viruses and CDKs is particularly intriguing. In some settings, viruses stimulate cell cycle progression or cyclin-CDK activity, whereas in others they suppress it. What determines whether a virus promotes or represses the cell cycle during active infection? Is there a “restriction point” beyond which viral manipulation becomes dispensable? Given the extensive crosstalk between stress-responsive CMGC kinases, such as p38 MAPK, and CDKs, addressing these questions will likely require integrated approaches combining stress-signaling paradigms with flow cytometry and single-cell omics technologies.

Finally, inhibitors targeting several CMGC kinases, including CDKs, p38 MAPK, and DYRKs, have been developed [12,219,247], with some already in clinical trials or even in use. However, off-target side effects remain a major limitation, likely reflecting the structural similarity among CMGC family members. Emerging strategies such as PROTACs (Proteolysis-targeting chimeras), which harness ubiquitin E3 ligases to induce selective protein degradation, offer a promising avenue to overcome these challenges and achieve greater specificity [279].

## Figures and Tables

**Table 1:** Functional impact/consequence of the interactions between selected viruses and CMGCs.

Virus(es)	CMGC Kinase Involved	Context-Dependent Function	Mechanism	Experimental System Used
HPV (DNA virus)	Cyclin/CDK	Likely proviral	- E6/E7 upregulates CyclinD1/CDK4, CyclinA/CDK2 and CyclinB/CDK1 complexes [58].	In vitro
		Likely proviral	- Through the abrogation of the DREAM pathway, HPV E7 downregulates DREAM target such as CDC25A, a suppressor of cyclin/cdk complex [59].	In vitro
		Likely proviral	- HPV-18 E1^E4 protein can interact with both cyclin E and cyclin A/CDK 2 via the RXL motif [60].	In vitro
HCMV (DNA virus)	Cyclin/CDK	Proviral	- CDK controls the expression of IE genes in the S/G2 phase [61].	In vitro
		Likely proviral	- HCMV upregulates Cyclin H [62].	In vitro
		Proviral	- vCDK/pUL97 and Host Factors CDK7–Cyclin H determine transcription pattern [63].	In vitro
		Proviral	- CDK8, positive regulator of CMV [64].	In vitro
HSV (DNA virus)	Cyclin/CDK	Antiviral	- S-phase cyclins downregulated by HSV1 ICP22/U_S_1.5 [65].	In vitro
		Proviral	- Inhibitors of CDK8 exhibit strong antiherpesviral activity [66].	In vitro
		Proviral	- Cdk1/2 inhibitor suppresses HSV1 replication [67].	In vitro
		Antiviral	- HSV-1 negatively regulates CDK9, inhibiting the phosphorylation of RNA polymerase II CTD [68].	In vitro
EBV (DNA virus)	Cyclin/CDK	Likely proviral	- Epstein–Barr Virus Nuclear Antigen 3C enhances cyclin A-dependent kinase activity by decreasing cyclin A/p27 association [69].	In vitro
		Proviral	- During lytic replication, EBV protein kinase, BGLF4, phosphorylate CDK inhibitor, p27Kip1, inducing its ubiquitin-mediated degradation [70].	In vitro
		Likely proviral	- EBNA3C suppresses cyclin/CDK inhibitors; p21WAF1/CIP1, p14ARF and p16INK4a [71].	In vitro and In vivo
		Likely proviral	- Cdc-2, cyclin E, CD23, and cyclin D2, are up-regulated as a result of EBV immortalization [72].	In vitro
		Likely antiviral	- EBV Zta induces the expression of CDKi, p21 and p27 causing G0/G1 cell cycle arrest and inhibiting cellular proliferation [73].	In vitro
VZV (DNA virus)	Cyclins	Likely proviral	- Upregulation of cyclins A, B1, and D3 during infection [74,75].	In vitro
KSHV (DNA virus)	Cyclin/CDK	Likely proviral	- Viral cyclin KSHV latent gene vCyclin (ORF72), a cellular Cyclin D2 homolog, drives KSHV-induced transformation potentially by disabling p27′s inhibitory function [76].	In vitro and In vivo
		Likely proviral	- Like V-cyclin, K-cyclin can complex with CDK6 and phosphorylate Rb and histone substrates in the G1 and S phases. K-cyclin can also interact with CDK9 and direct CDK9-driven p53 phosphorylation [77,78].	In vitro
Hepatitis B virus (DNA virus, but uses an RNA intermediate to replicate its genome)	Cyclin/CDK	Likely proviral	- Hepatitis B virus X protein activates G1-CDKs, does this by hypermethylating p16^INK4a^ promoter. p16 negatively inhibits CDK activity [79].	In vitro
		Likely proviral	- Another report showed that p53, a p21 activator, is repressed by this same protein [80].	In vitro
HPV (DNA virus)	DYRK	Likely proviral	- DYRK1A phosphorylates and stabilizes HPV16E7 oncoprotein [81].	In vitro
		Likely proviral	- HPV16 upregulates DYRK1A [82].	In vitro and clinical samples
Herpesviruses (DNA virus)	DYRK	Proviral	- DYRK1 inhibition exhibit strong antiherpesviral effect [83].	In vitro
		Likely proviral	- HCMV upregulates DYRK1A and B [84].	In vitro
Hepatitis B virus (DNA virus)	DYRK	Proviral	- DYRK1A interacts with the HBV genome and regulates transcription [85].	In vitro
		Antiviral	- DYRK4 inhibits HBV replication via autophagy [86].	In vitro
Pseudorabies virus (DNA virus)	DYRK	Proviral	- DYRK1A-dependent micropinocytosis promotes pseudorabies virus replication [87].	In vitro and In vivo
EBV (DNA virus)	p38 MAPK	Proviral	- EBV tegument protein BGLF2 activates p38 MAPK to promote viral reactivation in B cells [88].	In vitro
		Proviral	- EBV activation of p38 MAPK also promotes lytic replication in epithelial cells via Mnk1/2 activation (which is a substrate for p38 MAPK) [89].	In vitro
		Proviral	- EBV Z and R proteins promote p38 MAPK phosphorylation, together with JNK, which helps R disrupt viral latency [90].	In vitro
HCMV (DNA virus)	p38 MAPK	Likely proviral	- Following HCMV infection, p38 is phosphorylated and kinase activity is increased [91].	In vitro
KSHV (DNA virus)	p38 MAPK	Proviral	- KSHV induces p38 MAPK phosphorylation; inhibition affects GFP-tagged viral replication [92].	In vitro
		Proviral	- Also, in reactivation from latency models, p38 MAPK is required [93].	In vitro
HSV-1 (DNA virus)	p38 MAPK	Proviral	- A drug that blocks p38 MAPK activation suppresses viral replication [94].	In vitro
		Proviral	- HSV1 activates p38 MAPK, activation, dependent on ICP27 [95].	In vitro
		Proviral	- HSV1 activates p38 MAPK; inhibition negatively impacts viral gene expression [96].	In vitro
HBV (DNA virus)	p38 MAPK	Proviral	- HoxA10-mediated suppression of p38 MAPK reduces HBV replication and activity [97].	Clinical study
		Likely proviral	- p38 MAPK is activated by the HBV middle protein, upregulates IL6; might activate STAT3 to facilitate IL6 gene expression [98].	In vitro
Simian Cytomegalovirus (DNA virus)	GSK3	Likely proviral	- GSK3Β phosphorylates the assembly protein precursor of Simian Cytomegalovirus, promotes self-aggregation and interaction with major capsid protein [99].	In vitro
KHSV (DNA virus)	GSK3	Likely proviral	- LANA protein of KHSV binds to GSK3, allowing beta-catenin to accumulate and drive cells towards transformation [100].	In vitro
KHSV (DNA virus)	GSK3	Antiviral	- GSK3Β overexpression suppresses HSV1-induced KHSV reactivation [101].	In vitro
HSV and HCMV (DNA virus)	GSK3	Likely proviral	- HCMV upregulates GSK3Β in Alzheimer’s model [102].	In vitro
		Likely proviral	- HSV activates GSK3Β, induces synaptic dysfunction in cultured neurons [103].	Ex vivo
Human adenovirus (DNA virus)	GSK3	Proviral	- GSK3A promotes human adenovirus replication by phosphorylating viral L4-22K protein [104].	In vitro
HBV (DNA virus)	GSK3	Proviral	- GSK3A/B phosphorylates forkhead box K1/2 to drive HBV transcription [105].	In vitro
Adenovirus (DNA virus)	CLK	Likely proviral	- CLK1 regulates the alternative splicing of Adenovirus EA1 [106].	In vitro
HBV (DNA virus)	CLK	Proviral	- CLKs are activated during HBV infection in HepG2 cells; inhibition of these CLKs resulted in reductions in HBV intracellular cccDNA, pgRNA, and HBeAg titers [107].	In vitro
HPV16 (DNA virus)	SRPK	Proviral	- SRPK1 inhibition suppresses E2, E4 and L1 expression, affect late stages of HPV lifecycle [108].	In vitro
		Likely antiviral	- E1^E4 protein interacts with and inhibits SRPK1 [109], preventing it from phosphorylating E2, which is a viral replication and transcription factor [110].	In vitro
		Likely proviral	- HPV16 upregulates SRPK1 through E2 [111].	In vitro
HSV-1 (DNA virus)	SRPK	Likely antiviral	- SRPK1 is relocalized to the nucleus by HSV1 ICP27. This affects the ability of SRPK1 to phosphorylate SR proteins, and thus affects spliceosome assembly [112]; and inhibits splicing [113].	In vitro
VZV (DNA virus)	SRPK	Proviral	- IE4 interacts with and phosphorylates SRPK1, which is important for viral mRNA export [114].	In vitro
EBV (DNA virus)	SRPK	Proviral	- BZRF1 is phosphorylated by SRPK2, important for viral replication [115].	In vitro
HBV (DNA virus)	SRPK	Proviral	- SPRK binds to the CTD of HBV capsid protein, serving as a molecular chaperone that encourages accurate capsid assembly [116].	In vitro
		Antiviral	- SRPK1 and 2 suppress HBV replication by limiting the packaging efficiency of the pgRNA [117].	In vitro
		Proviral	- SPRK2 mediates the phosphorylation of HBV protein core and capsid assembly [118].	In vitro
		Proviral	- SPRK1 and 2 phosphorylate HBV core protein [119].	In vitro
SARS-CoV2 (RNA virus)	Cyclin/CDKs	Likely antiviral	- N protein of SARS-CoV-2 inhibits S phase progression; it does this by directly binding cyclin D, and inhibiting the Cyclin D/CDK4 complex [120].	In vitro
		Likely proviral	- Viral infection upregulates CDK1 kinase activity during the early infection phase; other CMGCs like p38/MAPK are also impacted [121].	In vitro
		Proviral	- CDK2 promotes viral RNA synthesis during SARS-CoV-2 infection [122].	In vitro
		Antiviral	- Cyclin D3 restricts the incorporation of SARS-CoV-2 envelope into virions [123].	In vitro
Influenza virus (RNA virus)	Cyclin/CDKs	Likely proviral	- Causes accumulation of cells in the G0/G1 boundary, thus preventing S-phase entry, accompanied by a reduction in cyclin D and E. Consistent in multiple influenza virus types [124].	In vitro
		Proviral	- CDK1 inhibitor functions as an antiviral drug against the flu [125]; similar results were obtained for the CDK9 inhibitor [126].	In vitro and In vivo
Avian reovirus p17 protein (RNA virus)	Cyclin/CDKs	Likely antiviral	- Reovirus p17 binds to CDK1, 2, 4, and 6, and negatively inhibits them or causes cytoplasmic retention. Also represses CAK activity by promoting p53–cyclin H interaction [127].	In vitro
HCV (RNA virus)	Cyclin/CDKs	Likely proviral	- Upregulation of cyclin D1 by HCV core via stat3 [128].	In vitro and In vivo
		Likely antiviral	- HCV suppresses CDK2/CAK complex [129].	In vitro
		Likely proviral	- In HCV patients, cyclin A, E, D1, CDK2 and CDK4 are upregulated [130].	Clinical study
		Likely antiviral	- Although other reports suggest upregulation of CDKi, p16 and p57 [131,132].	Clinical study
Coronavirus Infectious Bronchitis Virus (RNA virus)	Cyclin	Likely antiviral	- Downregulation of cyclins D1 and D2 during Coronavirus Infectious Bronchitis Virus infection, leads to G2/M accumulation [133].	In vitro
MHV (RNA virus)	Cyclin	Likely antiviral	- During MHV asynchronous infection, clear reduction in cyclins D1, D2, D3, and E [134].	In vitro
Infectious Bursal Disease Virus (RNA virus)	Cyclin	Proviral	- (CDK1)-cyclin B1 complex phosphorylates viral polymerase, VP1, and this facilitates viral replication [135].	In vitro
ZIKV (RNA virus)	Cyclin/CDKs	Likely proviral	- ZIKV activates CyclinA/CDK1, triggering mitotic entry in human neural progenitors [136].	In vitro
TBEV (RNA virus)	Cyclin/CDKs	Likely proviral	- Inhibition of all CDKs negatively impacts TBEV replication [137].	In vitro and In vivo
JEV (RNA virus)	Cyclin/CDKs	Proviral	- CDKi changes nucleolar morphology and negatively affects the distribution of JEV core protein, suppresses JEV and other flavivirus replication [138].	In vitro
SARS-CoV-2 (RNA virus)	p38 MAPK	Proviral	- Promotes p38/MAPK phosphorylation; inhibition of the pathway reduces viral replication [121].	In vitro
		Likely proviral	- SARS-CoV-2 E protein activates p38 MAPK [139].	In vitro and In vivo
		Likely proviral	- SARS-CoV-2 spike protein receptor binding domain interaction with ACE2 activates p-p38 MAPK, and this increases CHO sulfotransferases [140].	In vitro and In vivo
		Proviral	- p38β is an important host factor that promotes SARS-CoV-2 replication [141].	In vitro
Duck Tembusu virus (RNA virus)	p38 MAPK	Proviral	- Duck virus activates p38 MAPK; inhibition of this prevents robust viral titers [142].	In vitro
Bovine parainfluenza virus type 3 (RNA virus)	p38 MAPK	Likely proviral	- Upstream p38 MAPK signaling kinase, mkk3, upregulated by bovine parainfluenza virus [143].	In vitro
CHIKV (RNA virus)	p38 MAPK	Proviral	- Inhibition of p38 MAPK resulted in the downregulation of p-ERK through autophagy, and this represses CHIKV viral replication [144].	In vitro
		Proviral	- In macrophages, CHIKV induces p38 MAPK (and JNK) activation through NSP2 interaction; inhibition of this kinase suppresses viral replication [145].	In vitro
DENV (RNA virus)	p38 MAPK	Likely proviral	- DENV NSP1 activates p38 MAPK in endothelial cells; decreasing barrier integrity [146].	In vitro
		Likely proviral	- p38 MAPK inhibitor reduces the severity of DENV-induced liver injury [147].	In vivo
		Likely antiviral	- In another study, although p38 MAPK inhibition did not affect viral replication, it reduced virus-induced inflammatory response [148].	In vitro and In vivo
RSV and IAV (RNA virus)	p38 MAPK	Proviral	- RSV and flu virus replication are suppressed by the inhibition of p38 MAPK [149].	In vitro
Newcastle disease virus (RNA virus)	p38 MAPK	Proviral	- NDV activates p38 MAPK to enhance viral mRNA translation; NDV NP protein is implicated since it is important for cap-dependent translation [150].	In vitro
Reovirus (RNA virus)	p38 MAPK	Proviral	- p38 MAPK inhibitor impacts virus entry, capsid uncoating and post-uncoating event [151].	In vitro
HCV (RNA virus)	p38 MAPK	Proviral	- Triggers p38 MAPK activation by stimulating the interaction between p38α and TGF-β activated kinase 1 (MAP3K7) binding protein 1 (TAB1); advantageous for the virus, inhibitor prevents viral replication; this study also confirmed that the inhibition of p38 MAPK negatively affected SFTSV, HSV-1 and SARS-CoV-2 [152].	In vitro and clinical samples
		Likely proviral	- In combination with ethanol, HCV activates p38 MAPK in mouse models [153]	In vivo
		Likely antiviral	- By inhibiting Fas, HCV core protein suppresses p38 activation in HepG2 cells and transgenic mice [154].	In vitro and In vivo
Avian Reovirus (RNA virus)	p38 MAPK	Proviral	- AMPK activates p38 MAPK during avian reovirus infection, depletion or inhibition of AMPK caused reduced p38 MAPK phosphorylation; inhibition of p38 MAPK reduced viral replication [155].	In vitro
Influenza virus (RNA virus)	p38 MAPK	Likely antiviral	- During influenza virus infection in macrophages, inflammatory cytokines are produced, and this is dependent separately on p38 MAPK and IRF3 [156].	In vitro
		Likely proviral	- Inhibition of p38 MAPK reduced IFNβ production and rescues mice from lethal dose of H5N1 [157].	In vitro and In vivo
Encephalomyocarditis (EMC) virus (RNA virus)	p38 MAPK	Proviral	- In L929 cells, inhibition of p38 MAPK reduces viral replication [158].	In vitro
RSV (RNA virus)	p38 MAPK	Proviral	- A small hydrophobic protein of RSV phosphorylated by p38 MAPK, and this leads to its relocalization to the Golgi [159].	In vitro
Respiratory viruses (RNA virus)	p38 MAPK	Proviral	- p38 MAPK promotes the entry of respiratory virus, and this is dependent on TL4-MyD88 axis [160].	In vitro
Enterovirus (RNA virus)	p38 MAPK	Proviral	- Enterovirus 71 activates p38 MAPK, and this promotes viral replication [161].	In vitro
Junin Virus (RNA virus)	p38 MAPK	Proviral	- Junin virus, a new world arenavirus, activates p38 MAPK, inhibition of this protein reduces viral replication [162].	In vitro
Porcine epidemic diarrhea virus (PEDV) (RNA virus)	GSK3	Proviral	- Promotes the replication of PEDV by phosphorylating its nucleocapsid protein [163].	In vitro
Snakehead vesiculovirus (SHVV) (RNA virus)	GSK3	Proviral	- Phosphorylation of SHVV glycoprotein by GSK3 and p38 MAPK promotes viral replication [164].	In vitro
SARS-CoV-2 (RNA virus)	GSK3	Proviral	- Activated GSK3 phosphorylates the nucleocapsid of SARS-CoV-2, effect proviral [165]; possibly due to inhibitor pressure, viral mutant dispensable to GSK3β phosphorylation now identified [166].	In vitro and In vivo
		Proviral	- GSK3β interacts with and phosphorylates SARS-CoV-2 N protein [167].	In vitro
		Proviral	- Phosphorylates SARS-CoV N protein to promote viral replication [168].	In vitro
HTLV (RNA virus)	GSK3	Proviral	- HTLV infected T cells accumulates GSK3Β in the nucleus, effect proviral [169].	In vitro
HCV (RNA virus)	GSK3	Proviral	- When GSK3β inhibitor was used, HCV replication reduced [170].	In vitro
		Likely antiviral	- GSK3β phosphorylation of occludin possibly suppresses HCV entry [171].	In silico
		Proviral	- GSK3β enhances HCV replication by supporting miR-122 expression [172].	In vitro
DENV (RNA virus)	GSK3	Possibly antiviral	- DENV-2 inhibits glycogen synthase kinase 3 (GSK-3) and IL12 production [173].	In vitro
HIV (RNA virus)	GSK3	Proviral	- Upregulated in HIV-infected cells, targeting GSKβ abrogates HIV replication [174].	In vitro
		Proviral	- GSK3β inhibitor prevents Tat-Mediated HIV-1 replication [175].	In vitro
Coxsackievirus (RNA virus)	GSK3	Proviral	- Inhibiting GSK3β stabilizes β-catenin, suppresses the CPE of this virus [176].	In vitro
Influenza virus (RNA virus)	GSK3	Proviral	- GSK3βi suppresses virus replication [177,178].	In vitro and In vivo
		Proviral	- Inhibition of Akt suppresses GSK3β phosphorylation, viral replication reduced [179].	In vitro
Japanese encephalitis virus (RNA virus)	GSK3	Proviral	- Cyclin D stability during virus infection leads to reduced GSK3Β expression leading to JEV latency [180].	In vitro
Coronaviruses (RNA virus)	DYRK	Proviral	- DYRK1A positively regulates coronavirus (TGEV) replication through receptor upregulation [181].	In vitro
		Proviral	- DYRK1A promotes the entry of SARS-CoV-2, likely through the enhancement of chromatin accessibility [182].	In vitro
Sendai Virus (RNA virus)	DYRK	Possibly proviral	- DYRK2 represses Type I Interferon production during SeV infection [183].	In vitro
HIV (RNA virus)	DYRK	Antiviral	- DYRK1A through NFAT transcriptionally suppresses HIV-1 replication [184].	In vitro
		Antiviral	- DYRK1A modulates Cylin L levels, restricts HIV replication [185].	In vitro
HIV (RNA virus)	Cyclin/CDK	Proviral	- Tat-associated T-cell-derived kinase (TTK) drives the expression of Tat-dependent transcription of HIV-1 LTR. Tat induces the association of TTK with CDK2, and phosphorylates CTD of RNAPolII [186].	In vitro
		Proviral	- In HIV activation from latency model, CDK8 dissociates from the mediator complex, allowing TFIIH to be recruited, which then phosphorylates the RNAPol II CTD [187].	In vitro
		Proviral	- Inhibition of CDK2 and CDK6 block SAMHD1 phosphorylation, and negatively impacts HIV replication [188].	In vitro
		Proviral	- CDK11 associates with the subunits of TREX/THOC, which then recruits it to elongating RNAPII, allowing RNAPII CTD to be phosphorylated thus increasing viral gene transcription [189].	In vitro
		Proviral	- Tat interacts with cyclin-dependent kinase 13, increases HIV mRNA splicing [190].	In vitro
HTLV (RNA virus)	Cyclin/CDK	Likely proviral	- Expression of cyclin E/CDK2 upregulated during infection, possibly due to p27KIP1 suppression since it was reduced with Cyclin E/CDK2 upregulation [191].	In vitro
		Likely antiviral	- HTLV Tax suppresses the activity of the Cyclin A promoter, repression dependent on CREB/ATF binding [192].	In vitro
		Likely antiviral	- HTLV p30 can bind to cyclin A and cdk2, decreasing the complex formation, and preventing S phase entry [193].	In vitro
		Likely proviral	- Tax-dependent upregulation of cyclin E/CDK2 and cyclin D/CDK2 in HTLV infected cells through p21 and INK4 suppression [194].	In vitro
		Likely proviral	- HTLV stimulates the kinase activity of cyclin-dependent kinase 4 via binding to the N-terminus of HTLV-1 tax oncoprotein [195].	In vitro
HCV (RNA virus)	SRPK	Proviral	- inhibition of SRPK1/2 suppresses HCV gene expression and replication, as overexpression rescues [196].	In vitro
SARS-CoV-2 (RNA virus)	SRPK	Proviral	- Suppression of SRPK1/2 results in defective viral replication cycle, these kinases phosphorylate N protein [197].	In vitro
		Likely proviral	- Low level of SRPK2 in non-COVID patients as compared with COVID patients [198].	Clinical study
HIV and Sindbis virus (RNA virus)	SRPK	Proviral	- Inhibition of SRPK1 and 2 using SPRIN340 suppresses HIV and Sindbis virus replication [199,200].	In vitro
EBOLA (RNA virus)	SRPK	Proviral	- SRPK1 can phosphorylate the transcription factor of this virus VP30, and positively regulate viral gene expression [201].	In vitro
HIV (RNA virus)	CLK	Antiviral	- CLK1 and 2 have contrasting roles, depleting CLK1 increased HIV-1 promoter activity and gene expression [200].	In vitro
		Proviral	- CLK2 reduction suppressed HIV1 gene expression [200].	In vitro
		Proviral	- Inhibiting CLK2,3,4 using chlorhexidine blocked virus production [202].	In vitro
		Proviral	- Digitoxin, block all CLKs, inhibits the viral RNA processing [203].	Ex vivo
Influenza virus (RNA virus)	CLK	Proviral	- CLK1 is required for the synthesis of M2 RNA, a spliced variant of the M2 gene in Influenza virus [204]; CLK1 is important for the positive regulation of mRNA splicing in Influenza virus [205].	In vitro and In vivo
SARS-CoV-2 (RNA virus)	CLK	Likely proviral	- CLK1 identified among antiviral drug target for SARS-CoV2 [206].	In vitro
Sindbis virus (RNA virus)	CDKL5	Antiviral	- Depletion of CDKL5 reduced virophagy of Sindbis virus, as virus accumulates. Hence, CDKL5 is antiviral in this context [207].	In vitro and In vivo

## Data Availability

No new data were created or analyzed in this study. Data sharing does not apply to this article.

## Abbreviations

The following abbreviations are used in this manuscript:

AKT	Protein kinase B
ASK1	Apoptosis signal-regulating kinase 1
ATF2	Activating transcription factor 2
Bax	Bcl-2-associated X protein
CDKi	Cyclin-dependent kinase inhibitor
CHIKV	Chikungunya virus
CHOP	C/EBP homologous protein
CREB	cAMP response element-binding protein
CTD	C-terminal domain
DENV	Dengue virus
DLK	Dual leucine zipper kinase
DREAM	DP, RB-like, E2F, and MuvB complex
E2F	E2F transcription factor
EBV	Epstein–Barr virus
EGFR	Epidermal growth factor receptor
GPCR	G protein-coupled receptor
HBV	Hepatitis B virus
HBeAg	Hepatitis B e antigen
HCMV	Human cytomegalovirus
HCV	Hepatitis C virus
HIV	Human immunodeficiency virus
HPV	Human papillomavirus
HSV	Herpes simplex virus
HTLV	Human T-cell leukemia virus
IAV	Influenza A virus
ICK	Intestinal Cell Kinase, recently renamed CILK1
IRF3	Interferon regulatory factor 3
JEV	Japanese encephalitis virus
JNK	c-Jun N-terminal kinase
KSHV	Kaposi’s sarcoma-associated herpesvirus
LRP	Low-density lipoprotein receptor-related protein
MAK	male germ cell-associated kinase
MAP2K	Mitogen-activated protein kinase kinase
MAP3K	Mitogen-activated protein kinase kinase kinase
MEK	MAPK/ERK kinase
MEKK3	MEK kinase 3
MEKK4	MEK kinase 4
MHV	Mouse hepatitis virus
MK2/3	MAPK-activated protein kinase 2/3
Mnk1/2	MAPK-interacting kinase 1/2
MOK	MAPK/MAK/MRK-overlapping kinase
NAPA	N-terminal autophosphorylation accessory region
NF-κB	Nuclear factor kappa-light-chain-enhancer of activated B cells
NLK	Nemo-like kinase
PEDV	Porcine epidemic diarrhea virus
PEST	Proline, glutamate, serine, threonine-rich sequence
PI3K	Phosphoinositide 3-kinase
PPAR	Peroxisome proliferator-activated receptor
RAF	Rapidly accelerated fibrosarcoma kinase
RAS	Rat sarcoma small GTPase
RS domain	Arginine-serine-rich domain
RSV	Respiratory syncytial virus
RTKs	Receptor tyrosine kinases
Rb	Retinoblastoma protein
RdRp	RNA-dependent RNA polymerase
SAMHD1	SAM domain and HD domain-containing protein 1
SHVV	Severe acute respiratory syndrome coronavirus 2
STE20	Serine-glutamate-glycine motif
STAT3	Signal transducer and activator of transcription 3
TAB1	TGF-β-activated kinase 1-binding protein 1
TBEV	Tick-borne encephalitis virus
TCR	T-cell receptor
TDY motif	Threonine-aspartate-tyrosine motif
TEY motif	Threonine-glutamate-tyrosine motif
TQE motif	Threonine-glutamine-glutamate motif
TRAF	TNF receptor-associated factor
TXY motif	Threonine-any amino acid-tyrosine motif
VEGFR	Vascular endothelial growth factor receptor
VZV	Varicella-zoster virus
ZAP70	Zeta-chain-associated protein kinase 70
ZIKV	Zika virus
cMET	Mesenchymal–epithelial transition factor
cccDNA	Covalently closed circular DNA
dNTP	Deoxynucleotide triphosphate
eIF2B	Eukaryotic initiation factor 2B
mTOR	Mechanistic target of rapamycin
pgRNA	Pregenomic RNA
STE20	Sterile 20 Kinase
